# Assessing Dietary Habits of Saudi Patients With Type 2 Diabetes at Primary Healthcare Centers in Jeddah, Saudi Arabia

**DOI:** 10.7759/cureus.73333

**Published:** 2024-11-09

**Authors:** Abidah H AlEsawi, Abdullah Alsharif

**Affiliations:** 1 Department of Public Health, College of Medicine at Alfaisal University, Riyadh, SAU

**Keywords:** diabetes mellitus management, dietary, dietary control, health-care policy, holistic approaches, nutritional knowledge, nutritional therapy for diabetes, socio-economic factors, type 2 diabetic mellitus (t2dm)

## Abstract

Background

Type 2 diabetes mellitus (T2DM) is a prevalent chronic metabolic disorder characterized by impaired glucose regulation. Managing T2DM requires healthy dietary habits to achieve glycemic control and reduce the risk of complications. However, T2DM patients often struggle to adhere to these patterns due to cultural practices, socioeconomic factors, and insufficient nutritional education. Saudi Arabia has the second-highest prevalence of diabetes in the Middle East and ranks seventh in the world.

Aim

The current study aimed to evaluate dietary habits and assess the associated factors with them, such as sociodemographic and clinical characteristics.

Methods

This cross-sectional, observational, survey-based study was conducted between January and July 2023. Adult patients with T2DM attending primary healthcare centers in Jeddah Second Health Cluster, Saudi Arabia, were included in the study. Pediatric patients who are not Saudi and have type I diabetes or gestational diabetes mellitus were excluded from the study. The data were collected using a validated self-administered questionnaire, the UK Diabetes and Diet Questionnaire (UKDDQ). Statistical analysis was performed using the computer program IBM SPSS (version 26.0, IBM Corp., Armonk, USA). The total score of questions from question 1 to question 24 (except questions 18, 20, 22, and 23) was calculated for everyone regarding the validated questionnaire. Comparisons between the demographic factors and UKDDQ scores (not normally distributed) were conducted using the Mann-Whitney test. A Spearman’s test was done to explore the correlation between the numerical factors and the scores of the questions. The p-value less than 0.05 was considered statistically significant.

Results

The study included 428 Saudi patients with T2DM with a mean (standard deviation (SD)) age of 47.8 ± 11.7 years old. More than two-thirds were overweight 165 (38.6%) and obese 124 (29.0%). Two-thirds of the patients were married 278 (65%), and most were employed 322 (75.2%). The mean (SD) of the total UKDDQ score was 2.7 ± 0.4 out of 5. The results showed that males demonstrated better dietary habits than females, as reflected in their higher UKDDQ scores (P = 0.029). Additionally, patients with a university education or higher, as well as unemployed individuals, reported significantly better scores (P = 0.022 and P < 0.001, respectively). Furthermore, participants without a family history of diabetes had higher UKDDQ scores compared to those with such a history (P = 0.009). There was also a very weak positive correlation between age and UKDDQ scores (r = 0.135, P = 0.005). Conversely, a very weak negative correlation was found between Body Mass Index (BMI) and the number of years living with diabetes (r = -0.096), as well as between BMI and UKDDQ scores (r = -0.156), both with significant p-values (P = 0.049 and P = 0.001, respectively).

Conclusions

The findings underscore the complexity of factors influencing T2DM patients’ dietary habits. Notably, males and participants with lower BMI, higher education, unemployment, and those without a family history or complications exhibited more favorable dietary scores. Essential approaches include tailored interventions, health literacy initiatives, community programs, family involvement, long-term follow-up, multidisciplinary care, and addressing socioeconomic barriers are crucial to improve glycemic control and preventing diabetes-related complications.

## Introduction

Type 2 diabetes mellitus (T2DM) is a chronic metabolic disorder characterized by persistent hyperglycemia resulting from impaired insulin secretion or resistance [1]. Hyperglycemia is defined as blood glucose levels exceeding 125 mg/dL after fasting or 180 mg/dL two hours after a meal, and it can also be indicated by a random plasma glucose level of 200 mg/dL or higher accompanied by symptoms [2]. T2DM is much more prevalent (20 times) than type 1 diabetes [3]. The current global prevalence rate of diabetes mellitus is 6.1%, which ranks it among the top 10 causes of death and disability worldwide; almost all global cases (96%) are T2DM [4]. According to the World Health Organization (WHO), Saudi Arabia has the second-highest prevalence of diabetes in the Middle East and ranks seventh in the world [5]. The prevalence of T2DM is 32.8% and is expected to increase to 45.8% by 2030 in Saudi Arabia [6].

T2DM is a multifaceted condition arising from genetic, environmental, and metabolic risk factors. Age, obesity, and physical inactivity significantly enhance the likelihood of developing this condition, particularly in individuals with a family history of DM [7]. A study conducted among the Saudi population reported that physical inactivity, unhealthy diet, smoking, obesity, and aging are major risk factors for T2DM in Saudi Arabia.These factors face complex barriers, such as lack of education, social support, and a healthy environment [8]. Moreover, a systematic review found that dietary factors, including the increased consumption of processed and sugary foods like fast food and sugary beverages, are linked to a higher risk of T2DM in Saudi Arabia [9].

For managing T2DM, diet and lifestyle are crucial in controlling blood glucose levels [10]. Nutritional advice should be tailored to the individual and may involve limiting calorie intake for overweight patients, reducing the consumption of saturated fatty acids, and increasing the intake of unrefined carbohydrates. There are several ways to assess dietary habits, including traditional methods such as food records, food frequency questionnaires, 24-hour recalls, and screening tools. Nowadays, digital and mobile strategies that leverage technology are also available to enhance these traditional methods. Moreover, the UK Diabetes and Diet 10 Questionnaire compares food diaries to international nutritional recommendations for preventing and managing diabetes and helps evaluate important food habits [11].

Unfortunately, several traditional dietary patterns are common in Saudi Arabia, including the consumption of dates, desserts, and meat over rice dishes, which are rich in fat and carbohydrates [12]. A quarter of Saudi Arabia's population did not adhere to the recommended healthy diet, which includes consuming fruits and vegetables [13]. Furthermore, inadequate education, limited social support, and unfavorable environments may impede patients with T2DM from effectively managing their condition, where having knowledge and awareness of diabetes, its risk factors, complications, and management is essential for better disease control and improved quality of life [14]. Therefore, evaluating the dietary habits among patients with T2DM in Saudi Arabia is required to understand current practices, identify areas for improvement, and develop targeted interventions. Insufficient information from previous studies highlights the need for in-depth insights into T2DM patients' dietary behaviors in Jeddah. So, this study aimed to evaluate the dietary habits among patients with T2DM in Jeddah, Saudi Arabia, and to provide insights into improving dietary habits among T2DM patients.

## Materials and methods

Study settings and design

This was a cross-sectional observational study using a self-administered questionnaire survey. It was conducted in various primary healthcare centers (PHCs) located in the second health cluster of Jeddah, Saudi Arabia.

Study population

The sample size was calculated using the Raosoft online sample size calculator (Raosoft Inc., Seattle, USA). The margin of error was set at 5%, a confidence level of 95%, and maximum uncertainty (50% of the population with good eating habits) was considered [15]. Moreover, a minimum of 377 adult diabetic Saudi patients were needed to be included in the study. The study included Saudi T2DM patients aged 18 years and older who attended PHCs in Jeddah’s second health cluster, Saudi Arabia and were willing to participate in the study. While pediatric, non-Saudi, patients with type 1 diabetes mellitus (T1DM) or gestational diabetes mellitus (GDM), and those who refused to participate were excluded from the study.

Data collection method and tool

Data was collected from January to July 2023 using an anonymous self-administered questionnaire, adapted from the previously validated UK Diabetes and Diet Questionnaire (UKDDQ) and translated into Arabic to assess dietary habits among patients with T2DM [11]. Test-retest reliability and relative validity were conducted for the questionnaire, comparing food diaries to international nutritional recommendations for preventing and managing diabetes and helping evaluate main food habits.

The questionnaire had two parts: Section A included demographic characteristics of participants (age, gender, nationality, weight, height, marital status, employment status, family income, educational level, duration of T2DM, family history of diabetes, and the number of diabetic complications). Section B included 27 questions related to participants' dietary habits: 24 questions illustrating how often the participants eat different types of food and question 25 determining their concerns about their weight. Furthermore, questions 26 and 27 determine how important and confident they could change their diet.

Statistical analysis

Data were extracted into an Excel sheet (Version 2021, Microsoft Corporation, Redmond, USA) and then revised. The statistical analysis was performed using the computer program IBM SPSS (version 26.0, Armonk, NY, USA). Categorical variables were described in numbers and percentages. Continuous variables were reported as mean and standard deviation (SD) or median and interquartile range (IQR). A normality test was performed for all continuous variables. Comparisons between the demographic factors and UKDDQ scores (not normally distributed) were conducted using the Mann-Whitney test and the Kruskal-Wallis test. A Spearman’s test was done to explore the correlation between the numerical factors and the scores of the questions. Linear regression was used to investigate the confounding variable. A P-value less than 0.05 was considered statistically significant.

Regarding the scoring system, the total score of questions from question 1 to question 24 (except questions 18, 20, 22, and 23) was calculated for everyone regarding the validated questionnaire. The final score presented to the participant indicated the number of occurrences of A's, B's, C's, D's, E's, and F's they obtained out of a maximum of 20. Patients were categorized according to their dietary habits based on the total score. For instance, As and Bs mean healthy dietary habits, Cs and Ds mean less healthy, and Es and Fs mean unhealthy dietary habits. For the data analysis, the responses from each of the questionnaire items were re-coded into numerical values by applying the following codes: A=5, B=4, C=3, D=2, E=1, F=0. The mean UKDDQ score for each subject was then calculated from the 20 questionnaire scores, giving a final score ranging from 0 to 5. Higher scores indicated better dietary habits among the study subjects. Food diary coding and scoring were illustrated in the validated questionnaire [11].

Ethical consideration

Ethical approval was obtained from the Jeddah Health Local Ethics Committee in the Ministry of Health before the beginning of the study (approval number: A01549). Written informed consent was obtained from the participants. The data was collected anonymously. All information provided by the study participants was kept confidential and was not shared with anyone outside the research team. No personal identifiers or names were collected.

## Results

The study included 428 Saudi patients with T2DM who visited any PHCs in the second health cluster of Jeddah, Saudi Arabia, during the study period. Table 1 provides an overview of the patient's characteristics. The patients' mean (SD) age was 47.8 ± 11.7 years. The mean (SD) weight among participants was 48 ± 18 kg, and the mean (SD) height was 160.9 ± 9.5 cm. More than two-thirds were overweight 165 (38.6%) and obese 124 (29.0%). The patients had been diagnosed with diabetes with a mean age of 6.9 ± 6.5 years. Among the patients, 230 (53.7%) were males, while 198 (46.3%) were females. Regarding educational background, 187 (43.7%) had completed secondary education, and 100 (28.4%) had a university degree. Moreover, 278 (65%) of the patients were married. 322 (75.2%) were employed, and 363 (84.8%) had a family history of diabetes. Additionally, 222 (51.9%) of the participants had no diabetes-related complications. Furthermore, 248 (57.9%) participants had a family income of 10,000 SAR or more.

**Table 1:** Demographic characteristics of participants BMI: Body mass index, n: number, SD: standard deviation

Age (years)	Mean (SD)	47.8 (11.7)		
Parameters	Category	Count (n=428)		Percentage
Gender	Male	230		53.7
	Female	198		46.3
Marital status	Single	51		11.9
	Married	278		65
	Divorced	69		16.1
	Widowed	30		7
Educational level	Illiterate	28		6.5
	Primary school	89		20.8
	Secondary	187		43.7
	University	100		23.4
	Postgraduate	24		5.6
Employment Status	Employee	322		75.2
	Unemployed	106		24.8
Family income	Less than10,000 SAR	180		42.1
	10,000 SAR or more	248		57.9
Weight (Kg)	Mean (SD)	69.1 (12.7)		
Height (cm)	Mean (SD)	160.9 (9.5)		
BMI	Mean (SD)	26.9 (4.8)		
BMI categories	Normal weight	139	32.5	
	Overweight	165	38.6	
	Obese	124	29.0	
Family history of Diabetes	Yes	363	84.8	
	No	65	15.2	
Duration of diabetes (years)	Mean (SD)	6.92 (6.5)		
Number of diabetes-related complications	None	222	51.9	
	1	156	36.4	
	2	44	10.3	
	≥3	6	1.4	

**Figure 1:**
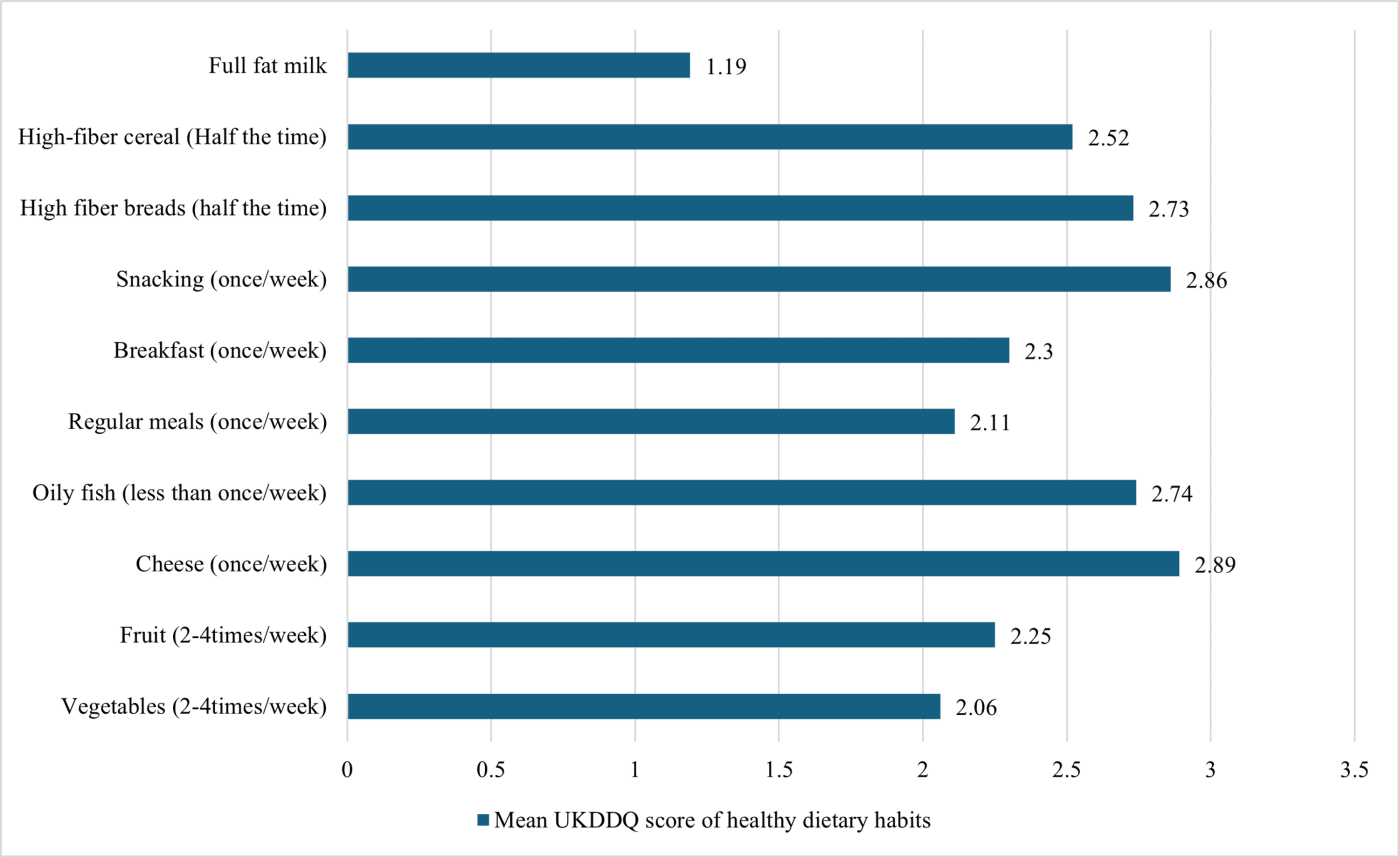
Mean UKDDQ score of healthy dietary habits UKDDQ: UK Diabetes and Diet Questionnaire

**Figure 2:**
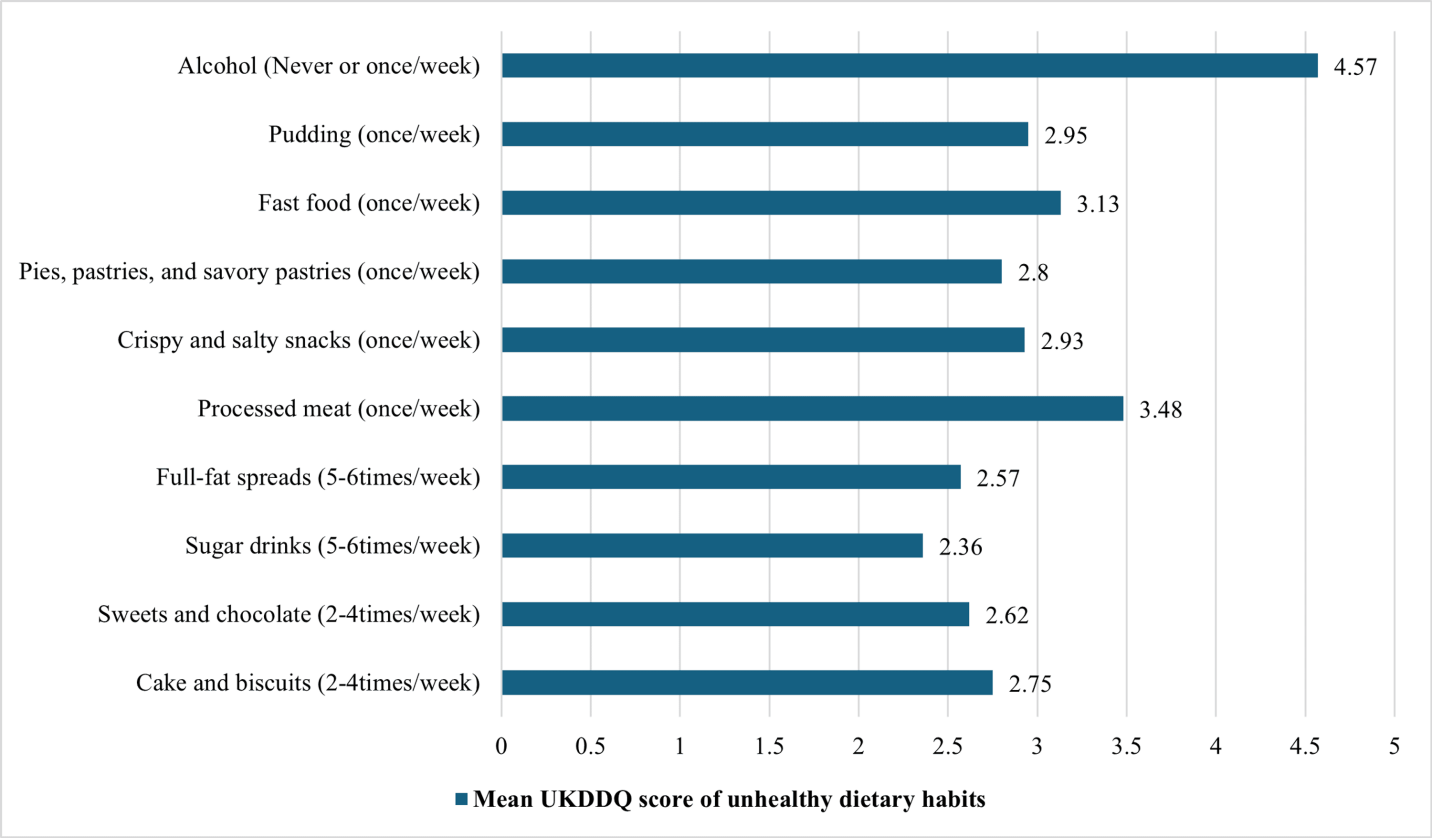
Mean UKDDQ score of unhealthy dietary habits UKDDQ: UK Diabetes and Diet Questionnaire

Table 2 shows the total UKDDQ score for each item in the questionnaire. The mean (SD) was 2.691 ± 0.430 out of 5. The choice “2-4 times a week” was selected for vegetables, fruits, cakes and biscuits, sweets and chocolate questions, with a mean (SD) of 2.06 ± 1.087, 2.25 ± 0.971, 2.75 ± 1.121, and 2.62 ± 1.131 out of 5, respectively. The answer “5-6 times a week” was selected for sugary drinks and full-fat spreads questions, with mean (SD) scores of sugary drinks and full-fat spreads of 2.36 ± 1.43 and 2.57 ± 1.212, respectively. Furthermore, the response “once a week” was selected for cheese, processed meat, crisps and salty snacks, pies, pasties, savory pastries, fast food, pudding, regular meals, breakfast, and snacking questions with a mean (SD) of 2.89 ± 0.964, 3.48 ± 1.14, 2.93 ± 0.969, 2.8 ± 1.052, 3.13 ± 1.116, 2.95 ± 1.05, 2.11 ± 1.078, 2.30 ± 1.124, and 2.86 ± 1.112, respectively. The answers “never or very rarely or less than once a week” were selected for alcohol questions with a mean (SD) of 4.57 ± 0.924, as shown in Figures 1 and 2.

**Table 2:** Scores of the UK Diabetes and Diet Questionnaire (UKDDQ) IQR: interquartile range, SD: standard deviation, UKDDQ: UK Diabetes and Diet Questionnaire

Vegetables	Mean (SD)	2.06 (1.087)
	95% confidence interval	1.96 - 2.16
	Median (IQR)	2 (2)
	Equivalent categorical score	2- 4 times a week
Fruit	Mean (SD)	2.25 (0.971)
	95% confidence interval	2.16 - 2.34
	Median (IQR)	2 (1)
	Equivalent categorical score	2- 4 times a week
Cakes and biscuits	Mean (SD)	2.75 (1.121)
	95% confidence interval	2.64 - 2.85
	Median (IQR)	3 (1)
	Equivalent categorical score	2- 4 times a week
Sweets and chocolate	Mean (SD)	2.62 (1.131)
	95% confidence interval	2.52 - 2.73
	Median (IQR)	3 (1)
	Equivalent categorical score	2- 4 times a week
Sugary drinks	Mean (SD)	2.36 (1.43)
	95% confidence interval	2.23 - 2.50
	Median (IQR)	2 (2)
	Equivalent categorical score	5 - 6 times a week
Full-fat spreads	Mean (SD)	2.57 (1.212)
	95% confidence interval	2.45 - 2.69
	Median (IQR)	2 (1)
	Equivalent categorical score	5 - 6 times a week
Cheese	Mean (SD)	2.89 (0.964)
	95% confidence interval	2.80 - 2.98
	Median (IQR)	3 (2)
	Equivalent categorical score	Once a week
Processed meat	Mean (SD)	3.48 (1.14)
	95% confidence interval	3.37 - 3.59
	Median (IQR)	3 (1)
	Equivalent categorical score	Once a week
Crisps and salty snacks	Mean (SD)	2.93 (0.969)
	95% confidence interval	2.84 - 3.02
	Median (IQR)	3 (2)
	Equivalent categorical score	Once a week
Pies, pasties, and savory pastries	Mean (SD)	2.8 (1.052)
	95% confidence interval	2.70 - 2.90
	Median (IQR)	3 (1)
	Equivalent categorical score	Once a week
Fast food	Mean (SD)	3.13 (1.116)
	95% confidence interval	3.03 - 3.24
	Median (IQR)	3 (2)
	Equivalent categorical score	Once a week
Pudding	Mean (SD)	2.95 (1.05)
	95% confidence interval	2.85 - 3.05
	Median (IQR)	3 (2)
	Equivalent categorical score	Once a week
Alcohol	Mean (SD)	4.57 (0.924)
	95% confidence interval	4.49 - 4.66
	Median (IQR)	5 (0)
	Equivalent categorical score	Never or very rarely/ Less than once a week
Oily fish	Mean (SD)	2.74 (0.062)
	95% confidence interval	2.62 - 2.87
	Median (IQR)	2 (2)
	Equivalent categorical score	Less than once a week
Regular meals	Mean (SD)	2.11 (1.078)
	95% confidence interval	2.01 - 2.21
	Median (IQR)	2 (2)
	Equivalent categorical score	Once a week
Breakfast	Mean (SD)	2.30 (1.124)
	95% confidence interval	2.19 - 2.40
	Median (IQR)	2 (1)
	Equivalent categorical score	Once a week
Snacking	Mean (SD)	2.86 (1.112)
	95% confidence interval	2.75 - 2.97
	Median (IQR)	3 (2)
	Equivalent categorical score	Once a week
High-fiber breads	Mean (SD)	2.73 (1.357)
	95% confidence interval	2.60 - 2.86
	Median (IQR)	3 (3)
	Equivalent categorical score	About half the time
High-fiber breakfast cereal	Mean (SD)	2.52 (1.555)
	95% confidence interval	2.38 - 2.67
	Median (IQR)	3 (3)
	Equivalent categorical score	About half the time
Type of milk	Mean (SD)	1.19 (1.825)
	95% confidence interval	1.02 - 1.37
	Median (IQR)	0 (3)
	Equivalent categorical score	Full fat
Total UKDDQ score	Mean (SD)	2.691 (0.430)
	95% confidence interval	2.6509 - 2.7327
	Median (IQR)	2.625 (0.5)

The gender, body mass index (BMI), educational level, employment, family history of diabetes, and diabetes-related complications factors had a statistically significant association with UKDDQ score with p-values of 0.029, 0.003, 0.022, <0.001, 0,002, and 0.009, respectively. The results found that males had a significantly (P=0.029) higher total UKDDQ score than females, with a mean (SD) of 2.7 ± 0.4 vs. 2.6 ± 0.4, respectively. Also, participants with normal weight had a marked (P=0.003) higher total UKDDQ score than overweight and obese patients, with a mean (SD) score of 2.8 ± 0.4 vs. 2.6 ± 0.4 and 2.7 ± 0.4, respectively. Additionally, patients with university or higher educational levels reported relatively significant (P=0.022) higher UKDDQ scores than others, with a mean (SD) score of 2.7 ± 0.4 vs. 2.6 ± 0.4, respectively. Moreover, unemployed patients reported a significantly (P<0.001) higher mean (SD) UKDDQ score than employed patients, 2.9 ± 0.5 vs. 2.6 ± 0.4, respectively. Patients with no family history of diabetes or diabetes-related complications reported significantly (P=0.002 and 0.009) higher total UKDDQ scores than others. Meanwhile, the other factors had no significant association with the total UKDDQ score, as shown in Table 3.

**Table 3:** Association between the individuals’ characteristics and UKDDQ score BMI: Body mass index, SD: standard deviation, UKDDQ: UK Diabetes and Diet Questionnaire; * P-value is significant at 0.05 level of significance

Factors		UKDDQ score (for all questions)	P- value
	Categories	Mean (SD)	
Age	< 40 years	2.7 (0.4)	0.476
	≥ 40 years	2.7 (0.4)	
Gender	Male	2.7 (0.4)	0.029*
	Female	2.6 (0.4)	
BMI	Normal weight	2.8 (0.4)	0.003*
	Overweight	2.6 (0.4)	
	Obese	2.7 (0.4)	
Educational level	University degree or higher	2.8 (0.5)	0.022*
	Less than a university degree	2.7 (0.4)	
Employment	Employed	2.6 (0.4)	<0.001
	Unemployed	2.9 (0.5)	
Marital status	Married	2.7 (0.4)	0.440
	Unmarried	2.7 (0.4)	
Monthly income	<10,000 SAR	2.7 (0.5)	0.218
	≥10,000 SAR	2.6 (0.4)	
Duration of diabetes	≤ 15 years	2.7 (0.4)	0.154
	> 15 years	2.9 (0.6)	
Family history of diabetes	Yes	2.7 (0.4)	0.002*
	No	2.8 (0.5)	
Diabetes-related complications	Yes	2.6 (0.4)	0.009*
	No	2.7 (0.5)	

There was a very weak positive significant correlation (r=0.135) between age and UKDDQ scores (P=0.005). Additionally, there was a very weak negative significant correlation between BMI and years of diabetes (r=-0.096 and -0.156, respectively) and UKDDQ scores (P= 0.049 and 0.001), respectively, as shown in Table 4.

**Table 4:** Correlation between age, BMI, years of diabetes, and UKDDQ scores BMI: Body mass index, UKDDQ: UK Diabetes and Diet Questionnaire; *P-value is significant at 0.05 level of significance

Factors	UKDD score		P-value
Age	Correlation coefficient	0.135	0.005*
BMI	Correlation coefficient	-0.096	0.049*
Years of diabetes	Correlation coefficient	-0.156	0.001*

Table 5 illustrates that 11.1% of this study's results could truly and reliably reflect the influence of age, gender, employment status, and diabetes-related complications on UKDDQ scores by applying linear regression analysis. Age, male gender, and unemployment status had a significant positive effect on high UKDDQ scores (β = 0.007, P<0.001; β = 0.114, P=0.006; and β = 0.194, P<0.001; respectively), while diabetes-related complications had a significant negative effect on sleep quality (β = −0.090, P=0.042).

**Table 5:** Linear regression analysis of UKDDQ scores BMI: Body mass index; UKDDQ: UK Diabetes and Diet Questionnaire; * P-value is significant at 0.05 level of significance

Factors	Unstandardized coefficients B		95%CI	P-value
Age	0.007		[0.003-0.010]	<0.001*
BMI	-0.007		[-0.016-0.002]	0.110
Years of diabetes	0.001		[-0.009-0.009]	0.987
Gender (Reference: Female)	0.114		[0.032-0.195]	0.006*
Educational level (Reference: Less than university degree)		0.074	[-0.016-0.163]	0.106
Employment (Reference: Employed)		0.194	[0.100-0.289]	<0.001*
Family history of diabetes (Reference: No)		-0.082	[-0.194-0.031]	0.154
Diabetes-related complications (Reference: No)		-0.090	[-0.177—0.003]	0.042*

## Discussion

Among the countries suffering from this diabetes epidemic, Saudi Arabia shows a high prevalence rate with a percentage of 23.1% [16]. Diabetic patients' dietary habits refer to food choices and consumption patterns based on nutritional advice provided by healthcare providers [17]. Studies have also shown that effectively controlling the dietary habits of patients with diabetes can lead to improvements in glycated hemoglobin (HbA1c) levels, thus helping to delay the onset of diabetes-related complications [18]. In Gulf countries, particularly in Saudi Arabia, there is a lack of effective dietary habits programs aimed at preventing and controlling complications arising from non-communicable diseases, such as T2DM, that are related to diet [19]. The main goal is to inform culturally appropriate guidelines and improve management strategies. Therefore, this study aimed to examine the dietary habits of patients with T2DM.

It is known that undesirable glycemic control mainly results from low nutritional knowledge and non-compliance with a healthy lifestyle, which is essential for patients with T2DM in the short and long term [20]. In the present study, patients showed a moderate level of maintaining healthy dietary habits and reported a mean (SD) total UKDDQ score of 2.691 ± 0.43 out of 5. A previous study conducted among South Asian men found that the participants had a slightly higher mean (SD) total UKDDQ score of 3.44 ± 0.43 [21]. Another study in Saudi Arabia found that the dietary habits among T2DM patients were poor [22]. In the current study, several factors attributed to the total UKDDQ score, including age, BMI, diabetic duration, gender, educational level, employment status, family history of diabetes, and diabetes-related complications. According to our results, males showed healthier behaviors and reported higher scores than females. Similarly, a study proved that gender has been recognized as a critical factor that significantly impacts lifestyles and gender differences have been associated with dietary habits and individual responses to dietary intake [23].

In addition, there was a positive relationship between age and total UKDDQ score. Another study found that older participants tend to have healthier dietary habits than younger ones [24]. However, there was a converse correlation between diabetes duration and the total UKDDQ score among our subjects. It seems that patients who had longer diabetic duration had a higher awareness level regarding healthy diet consumption compared to those recently diagnosed. Obviously, those with a longer duration may gain great experience in dealing with their condition. Moreover, participants with higher educational levels showed healthier behavior than those with low educational levels. This may be attributed to the fact that education can influence a person's understanding of diabetes, self-management skills, and access to resources [25].

Regarding dietary habits, most patients in our study reported eating vegetables and fruits two to four times per week, with an inadequate mean score of (2.06 and 2.25, respectively). On the difference, another study found that participants followed healthier behavior and noted that the mean vegetable and fruit scores were 3.65 and 3.51, respectively [21]. However, a Saudi study found that the participants consume vegetables and fruits five to six times per week [22]. This is explained by the unhealthy dietary habits of Arab populations rich in fat and processed foods [19], while dietary fiber consumption is low [26].

Many studies have specifically reported a positive relationship between the intake of sugars and the development of T2DM [27]. In the present study, patients consumed cakes, biscuits, sweets, and chocolates two to four times per week and reported a mean score of 2.75 and 2.62, respectively. Our participants' fast food intake rate was once a week, with an adequate mean score (3.13 out of 5). A previous study in Lebanon found a direct association between desserts and fast-food patterns with T2DM [28].

According to oily fish intake, it is suggested that consumption of oily fish is related to a lower risk of T2DM. In addition, fish oil supplements, especially constant use over time, have a relationship with a lower risk of T2DM [29]. Our participants seem unaware of the benefits of oily fish, and most of them reported that they eat oily fish less than once a week. Our results are similar to those of an Asian study, which reported that most individuals indicated low awareness of the benefits of oily fish as they consume oily fish less than once a week [21].

Over the past ten years, the trend of skipping breakfast has dramatically aggravated among children, adolescents, and adults [30]. In addition, it is estimated that skipping breakfast is associated with overweight and other health issues [30]. On the other hand, most participants in the current study indicated that they ate breakfast once a week. Another study conducted in Tabuk, Saudi Arabia found that breakfast skipping was higher among T2DM patients compared to controls (P<0.001) [20]. Our findings in Jeddah were unlike those of a study with different populations, indicating that the subjects followed a healthy behavior and ate breakfast daily [21].

Limitations

This study has several limitations, primarily stemming from its reliance on self-reported dietary data, which introduces the potential for recall bias and social desirability bias. The focus on a specific population in Saudi Arabia may limit the generalizability of the findings to other regions or diverse groups. Additionally, the cross-sectional design restricts the establishment of causal relationships between dietary habits and diabetes management. The study also did not account for confounding variables, limiting the analysis to bivariate associations. While the survey explored various aspects of dietary habits, it provided limited insight into specific dietary factors, such as oily fish and alcohol consumption. Future research should investigate these aspects to better understand their impact on T2DM management.

Recommendations

Future research should evaluate the effectiveness of interventions targeting dietary habits among individuals with T2DM, particularly in diverse populations from different regions in Saudi Arabia. Incorporating objective measures, such as dietary logs or biomarker assessments, could enhance the reliability and generalizability of findings. Advanced research methodologies, including machine learning and data mining techniques, would allow for a more nuanced analysis of the complex relationships between demographic factors and dietary habits. Additionally, broadening the research scope to include other lifestyle factors not covered in this study could provide a more comprehensive understanding of influences on health behaviors.

## Conclusions

In conclusion, this study investigated the dietary habits of patients with T2DM in Saudi Arabia. The findings indicated that participants exhibited moderate levels of healthy dietary habits, as measured by the UKDDQ. Key factors influencing dietary habits included male gender, lower BMI, higher education levels, unemployment status, and the absence of a family history of diabetes or complications, all of which were associated with more favorable dietary scores. Despite these insights, the study revealed that the consumption of vegetables, fruits, and oily fish fell below the recommended levels, while the intake of sugary foods, sweets, and full-fat spreads was relatively high. These dietary patterns highlight the need for targeted interventions to improve the dietary habits of individuals with T2DM. Enhancing dietary habits is essential for achieving optimal glycemic control and reducing the risk of diabetes-related complications.
